# Comparable Number of Genes Having Experienced Positive Selection among Great Ape Species

**DOI:** 10.3390/ani11113264

**Published:** 2021-11-15

**Authors:** Duo Xie, Guangji Chen, Xiaoyu Meng, Haotian Wang, Xupeng Bi, Miaoquan Fang, Chentao Yang, Yang Zhou, Erping Long, Shaohong Feng

**Affiliations:** 1 College of Life Sciences, University of Chinese Academy of Sciences, Beijing 100049, China; chenguangji@genomics.cn; 2 BGI-Shenzhen, Shenzhen 518083, China; bixupeng@genomics.cn (X.B.); fmquan3014@outlook.com (M.F.); yangchentao@genomics.cn (C.Y.); zhouyang@genomics.cn (Y.Z.); 3 State Key Laboratory of Genetic Resources and Evolution, Kunming Institute of Zoology, Chinese Academy of Sciences, Kunming 650223, China; mengxiaoyu@mail.kiz.ac.cn (X.M.); wanghaotian@mail.kiz.ac.cn (H.W.); 4 Kunming College of Life Science, University of Chinese Academy of Sciences, Kunming 650223, China; 5 Laboratory of Translational Genomics, Division of Cancer Epidemiology & Genetics, National Cancer Institute, National Institutes of Health, Bethesda, MD 20850, USA; erping.long@nih.gov

**Keywords:** great ape, evolution, adaptive evolution, positive selection gene, comparative genomics

## Abstract

**Simple Summary:**

It is of great interest to quantify adaptive evolution in human lineage by studying genes under positive selection, since these genes could reveal insights into our own adaptive evolutionary history compared to our closely related species and often these genes are functionally important. We used the great apes as the subjects to detect gene-level adaptive evolution signals in all the great ape lineages and investigated the evolutionary patterns and functional relevance of these adaptive evolution signals. Even the differences in population size among these closely related great apes have resulted in differences in their ability to remove deleterious alleles and to adapt to changing environments, we found that they experienced comparable numbers of positive selection. Notably, we identified several genes that offer insights into great ape and human evolution. For example, *SOD1*, a gene associated with aging in humans, experienced positive selection in the common ancestor of the great ape and this positive selection may contribute to the aging evolution in great apes. Overall, an updated list of positively selected genes reported by this study not only informs us of adaptive evolution during great ape evolution, but is also helpful to the further study of non-human primate models for disease and other fields.

**Abstract:**

Alleles that cause advantageous phenotypes with positive selection contribute to adaptive evolution. Investigations of positive selection in protein-coding genes rely on the accuracy of orthology, models, the quality of assemblies, and alignment. Here, based on the latest genome assemblies and gene annotations, we present a comparative analysis on positive selection in four great ape species and identify 211 high-confidence positively selected genes (PSGs). Even the differences in population size among these closely related great apes have resulted in differences in their ability to remove deleterious alleles and to adapt to changing environments, we found that they experienced comparable numbers of positive selection. We also uncovered that more than half of multigene families exhibited signals of positive selection, suggesting that imbalanced positive selection resulted in the functional divergence of duplicates. Moreover, at the expression level, although positive selection led to a more non-uniform pattern across tissues, the correlation between positive selection and expression patterns is diverse. Overall, this updated list of PSGs is of great significance for the further study of the phenotypic evolution in great apes.

## 1. Introduction

Adaptive evolution has been reported to be associated with many phenotypic changes in humans [1,2,3]. The Hominid, known as the great ape, has experienced various adaptive evolutionary innovations, such as significant sexual dimorphism [4], increased body mass [5], and increased brain volume correlated to high-order cognitive ability [6]. Identifying genes targeted by adaptive evolution (also known as Positively Selected Genes, PSGs) will advance our understanding of the underlying genetic basis of evolution. Although many studies have investigated PSGs in humans or primates, the detection of positive selection relies on the accuracy of orthology, the quality of assemblies, models, and alignments [7,8,9,10]. The improved branch-site model built in PAML is a commonly used model to detect positive selection [11]. In the branch-site model, branches in the tree are classified into foreground branches, in which some sites have been targeted by positive selection (the dN/dS of sites > 1), and background branches, in which no positive selection occurs [12]. Then an LRT is performed to compare an alternative model in which some sites undergo positive selection on the foreground branches with a null model that does not. Bakewell and colleagues [13] used this improved branch-site model to identify PSGs in the human and the chimpanzee, with rhesus as the outgroup. They performed analysis based on old, less-complete genome assemblies without filtering for the low confidence PSG candidates, which will cause potential false negatives. Lee et al. [14] performed the latest genome-scale positive selection test in primates to identify high-confidence PSGs with a stringent threshold. However, their analysis was based on the site model, which cannot detect the branch-level positive selection signals [15], and they did not use the latest upgraded genome assemblies [16].

In this study, utilizing the orthologs and gene families produced by the latest genome assemblies and gene annotations, we searched for high-confidence positive selection across all sites of all orthologous genes in all great ape lineages, and showed that the great ape species experienced comparable numbers of positive selection; even the differences in population size among these closely related great apes have resulted in differences in their ability to erase deleterious alleles and to adapt to changing environments. We also observed that the majority (63%) of the PSGs are from multiple-gene families, suggesting various positive selection resulted in the functional divergence of duplicates. Based on these PSGs, we investigated their functional contribution to great ape evolution and their expression pattern. We also tested human disease adaptations among our PSGs. Overall, this study provides a timely update of PSG candidates that might have contributed to the adaptive evolution of great apes.

## 2. Materials and Methods

### 2.1. Identification of Orthologs in Six Primates

We applied Orthofinder [17] among six primates and identified 14,758 one-to-one orthologs (Table 1). After excluding 361 ortholog groups containing gene models with in-frame stop codon or whose length of CDS was not a multiple of three, we retained 14,397 one-to-one orthologs and used them in downstream analysis. The six primate genomes included were human (*H. sapiens*, GRCh38.p13), chimpanzee (*P. troglodytes*, Clint_PTRv2), gorilla (*G. gorilla*, Kamilah_GGO_v0), orangutan (*P. abelii*, Susie_PABv2), gibbon (*N. leucogenys*, Asia_NLE_v1), and rhesus (*M. mulatta*, Mmul_10).

To ensure the coherence of the annotation resource, we collected all the corresponding annotations from the NCBI database based on the latest assembly version (Table 1). We downloaded respective NCBI gene sets and kept the longest transcript for each protein-coding gene. For humans, we only kept the protein-coding genes from primary assembly to avoid redundant gene models.

### 2.2. Building the Gene Family

To determine the gene families, we extracted coding sequences (CDSs) according to the gene annotations and genomes of corresponding species and then translated these coding sequences into protein sequences. Next, we conducted an all-against-all blastp search (blast-2.2.26; E-value < 10^−7^) [18] of these protein sequences in the six primate species [19]. Finally, protein sequences were clustered into gene families based on identity using hcluster_sg (hcluster_sg -m 750 -w 0 -s 0.34 -O) in Treefam. This gene family information was used to classify orthologous groups into different gene families. We classified gene families with multiple members from one or more species as multigene families, and gene families with only single-copy genes as single-gene families.

### 2.3. Positive Selection of Genes

We screened orthologous genes to explore the patterns of positive selection in great apes: *H. sapiens*, *P. troglodytes*, Hominini, *G. gorilla*, Homininae, *P. abelii*, and Hominidae. We first extracted the CDS from genomes based on protein-coding gene annotations and translated CDS into protein sequences. We then aligned these protein sequences using GUIDANCE (guidance.pl --program GUIDANCE --seqType aa --msaProgram PRANK --MSA_Param “\+F “; v2.02) [20], back-translated protein alignments into CDS alignments based on the original CDS, and fed these as the input to the improved branch-site model in PAML software package v4.9j [11,12]. We labeled all terminal branches and inner branches of great apes as foreground branches separately to perform multiple branch-site model tests for each ortholog group. To exclude potential artifacts, the final PSGs were determined by the following steps: (1) *p*-values were computed using the LRT test based on the output from PAML software and only genes with *p*-value < 0.05 were regarded as PSG candidates and used in the following analysis; (2) the PSG candidates with potential sites for selection (Bayes Empirical Bayes (BEB) posterior probability > 0.95) with gaps in 5 upstream or downstream amino acids were filtered out to exclude the false positive PSGs caused by alignment gaps (see an example in Figure S1); (3) filtered out PSG candidates with positively selected sites whose GUIDANCE alignment column score (range from 0 to 1) was less than 1.0 (the highest column score that ensures high-quality column alignment), (4) and excluded the false positive PSGs potentially caused by genetic drift (K-value < 1 and *p*-value < 0.05) by running RELAX from the Hyphy package in PSG candidates [21].

### 2.4. Population Analysis of PSGs

We used population SNPs datasets (in VCF format) of great ape species to investigate whether the positively selected sites of PSGs were fixed in these lineages. For humans, chimpanzees, and orangutans, we downloaded their corresponding SNPs dataset from the Ensembl database (Ensembl release 104). For gorillas, whose SNPs dataset was not available in the Ensembl database, we downloaded the published data by Prado-Martinez et al. [22]. Since the reference genome assemblies of chimpanzee, gorilla, and orangutan SNPs datasets were different from the assemblies used in this study, we converted the coordinates of these VCF files by LiftoverVcf in GATK (v4.1.4.1) based on the chain files between different assemblies produced by LASTZ [23] followed by chaining and netting [24] using scripts from the UCSC genome browser source code.

We defined a positively selected amino acid site as fixed in a population if all the allele frequency of these alleles located in this codon > 0.95 (in humans whose allele frequency information was available) or it has no alternative allele in this codon (in chimpanzees, gorillas, and orangutans where the allele frequency information was not available). A PSG was regarded as fixed if it contained at least one fixed positively selected amino acid site.

### 2.5. Expression Analysis of PSGs

To investigate the expression pattern of PSGs, we used gene expression profiles (Strand-specific RNA-seq of 13 human tissues from Michael Snyder’s lab for the ENCODE project) in the Expression Atlas database (https://www.ebi.ac.uk/gxa/home, last accessed 18 April 2021). We used log_2_(TPM + 1) as the proxy to measure the differences in the expression patterns (tissue specificity or expression level) of PSGs and their most similar paralogous non-PSGs, which were paralogs with the highest similarity to PSGs. We compared tissue specificity and expression level of the human gene expression pattern between PSGs and non-PSGs. We used the putative τ [25] as a proxy of the tissue specificity of a gene and used mean expression level across all tissues as a proxy of expression level.

### 2.6. Association Analysis of PSGs in Great Apes

To test whether PSGs tend to be associated with disease, we downloaded disease-associated genes from OMIM [26] and performed all the statistical analysis with R.

## 3. Results

### 3.1. Comparable Number of Genes Had Experienced Positive Selection among Great Ape Species

**Figure 1:**
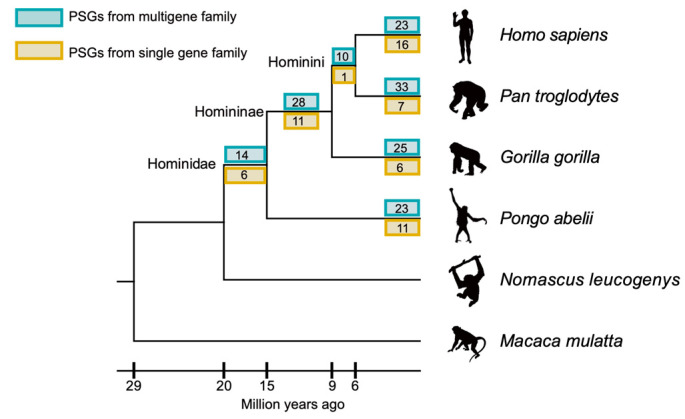
The number of PSGs in each great ape lineage. Blue and yellow numbers show the number of PSGs in multigene families and single-gene families, respectively. The times of branches come from the TimeTree website (http://www.timetree.org/, last accessed 3 July 2019). Animal illustrations are from phylopic.org, and are credited to NASA (*H. sapiens*, https://creativecommons.org/publicdomain/mark/1.0/, last accessed 23 July 2019), Gareth Monger (*P. abelii*, https://creativecommons.org/licenses/by/3.0/, last accessed 23 July 2019), Michael Keesey (*G. gorilla*, https://creativecommons.org/publicdomain/zero/1.0/, last accessed 23 July 2019), T. Michael Keesey and Tony Hisgett (*P. troglodytes*, https://creativecommons.org/licenses/by/3.0/, last accessed 23 July 2019).

The orthologous relationships of all gene families allowed us to investigate the forces of positive natural selection on genes derived from the common ancestors in different great ape lineages. Using an improved branch-site likelihood method [11], we searched for positively selected genes (PSGs) at all evolutionary branches in great apes. We first identified 14,397 one-to-one orthologous genes that evolved from the common primate ancestor using the gibbon and the rhesus as the outgroup. After filtering out false positives potentially caused by alignment error and genetic drift (Materials and Methods), our analyses revealed that 211 orthologs had experienced positive selection in at least one evolutionary branch (Table 2 and Table S1). Of these genes, 208 were positively selected at one evolutionary branch and 3 were positively selected in two evolutionary branches. Overall, the great ape species harbor comparable PSGs (31~40 PSGs): from 31 PSGs in gorillas (the fewest among great ape lineages), 39 PSGs in humans, to 40 PSGs in chimpanzees (the largest among the four tested extant great ape species) (Table 2; Figure 1). Compared to terminal branches, the inner branches have fewer PSGs. Based on the published population SNPs datasets (Materials and Methods) [22,27], we found most of the PSGs (204 out of 211 PSGs) contain at least one amino acid changes that have been fixed in the population (454 out of 484 positively selected sites, Table 3).

Bakewell et al. 2007 used the branch-site likelihood model but old annotation and orthologous data, and identified 154 and 233 PSGs in humans and chimpanzees, respectively [13]. These numbers decreased to 39 in humans and 40 in chimpanzees in our analyses. We found that 11 human PSGs and 9 chimpanzee PSGs have been reported by previous study. Many of the PSGs (77/154 in the human and 97/233 in the chimpanzee) could be attributed to the different input caused by different annotation versions and ortholog assignment and problematic gene models (Table 4). Among these, 58 PSGs in humans and 75 PSGs in chimpanzees in the previous study were not listed in the current annotations. Additionally, several previously reported PSGs (19 in humans and 25 in chimpanzees) were not included in our orthologs. This was because of different species sampling (16 in humans and 22 in chimpanzees) and low confidence gene models with in-frame stop codon or the length of CDS was not a multiple of three in these ortholog groups (three in humans and three in chimpanzees) and thus these orthologs were not included in this analysis.

Beyond different input concerns, 66 human PSGs and 124 chimpanzees PSGs in Bakewell’s list were not identified as no PSGs in this study because of different data processing method. Specifically, a total of 25 human PSGs and 96 chimpanzee PSGs did not pass our LRT test. Moreover, 38 human PSGs and 27 chimpanzee PSGs on Bakewell’s list did not contain any positively selected sites with BEB posterior probability greater than 0.95. Next, 3 PSGs in humans and 27 PSGs in chimpanzees in Bakewell’s list were filtered out because their positively selected sites were biased. Apart from these, we detected 25 human PSGs and 30 chimpanzee PSGs which were not identified by Bakewell et al. This may be caused by the more complete assembly or our denser species sampling and thus increases the power of detection of PSGs.

We observed that 73% (154/211) of PSGs were from multigene families (Figure 1). In principle, once a new gene is duplicated from its ancestral copy, especially by DNA level duplication in which often provides a functional promoter to express the duplicated gene [28], it introduces a redundant function to the parental copy, which would result in relaxed selective constraint in one copy and be ultimately lost through pseudogenization in most cases [29]. Thus, we investigated whether genes with duplicated copies are less likely to be positively selected by testing whether PSGs are less enriched in multiple-gene families. We found the number of PSGs in multiple families was not significantly less than PSGs in single-gene families (*p*-value range from 0.186 to 0.992) (Table 5), suggesting imbalanced positive selection resulted in the functional divergence of duplicates. These results are consistent with the hypothesis that gene duplication events can also offer new genetic materials for selection [30].

### 3.2. The PSGs Contributed to Functional Evolution of Great Ape

To explore how PSGs contribute to functional evolution in great apes, we first performed gene enrichment analyses with Metascape [31], and found that PSGs in humans and ancestral great ape lineage are enriched in essential biological functions such as positive regulation of cell junction assembly and the superoxide metabolic process (Figure S2). We then investigated the functional contributions of PSGs by focusing on specific genes in great apes. We found that 12 SLC genes were positively selected in great apes. For example, *SLC39A6*, a gene of the SLC39 gene family, was positively selected in humans. SLC39 transporters primarily serve to pass zinc into the cytoplasm and play critical roles in maintaining cellular zinc homeostasis [32]. Homozygous knockout of SLC39 family genes cause neurodegeneration growth retardation, morphological defects, and abnormal neurogenesis in mice [33,34]. The positive selection of *SLC39A6* may be associated with the distinct neurogenesis in humans and correlated to high-rank cognition ability [35,36]. We also found two leukocyte antigens (*CD36* and *CD3E*) under positive selection in the common ancestor of the great ape. Interestingly, *CD36* has been reported related to malarial resistance in humans [37] and mutations in *CD36* are associated with malaria susceptibility [38] and protection against malaria [39], indicating that the positive selection of *CD36* may be associated with malarial resistance evolution in great apes, although the difference in malarial resistance between great ape and other primates has yet to be investigated.

**Figure 2:**
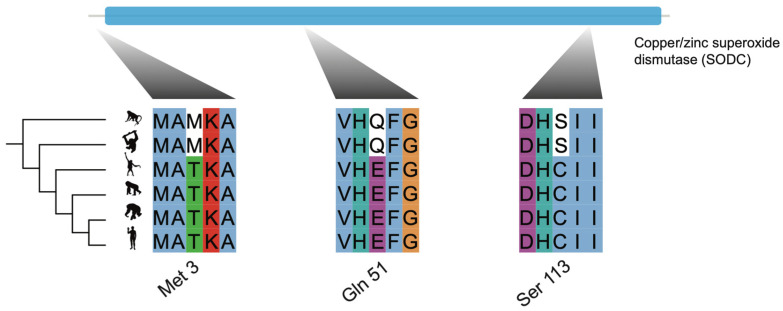
Positive selection targeting on *SOD1* in the common ancestor of great apes. The top blue bar denotes the functional domain of *SOD1*. *SOD1* experienced positive selection at multiple sites (Met 3, Gln 51, Ser 113). Animal illustrations are from phylopic.org, and are credited to NASA (*H. sapiens*, https://creativecommons.org/publicdomain/mark/1.0/, last accessed 23 July 2019), Gareth Monger (*P. abelii*, https://creativecommons.org/licenses/by/3.0/, last accessed 23 July 2019), Michael Keesey (*G. gorilla*, https://creativecommons.org/publicdomain/zero/1.0/, last accessed 23 July 2019), T. Michael Keesey and Tony Hisgett (*P. troglodytes*, https://creativecommons.org/licenses/by/3.0/, last accessed 23 July 2019).

We found four PSGs that may be associated with aging in great apes. For example, *SOD1*, which encodes superoxide dismutase 1, responsible for destroying free superoxide radicals in the body [40], was detected to have been targeted by positive selection in the common ancestor of the great apes at multiple sites (Met 3, Gln 51, Ser 113; Figure 2). *SOD1* contributes to the senescence as an important player in cellular senescence by catalyzing superoxide radicals (O_2_^._^) to H_2_O_2_ and O_2_ [41] and mediating the p53 pathway [42], which are both involved in the cellular senescence process [43], and the overexpression of this gene in fruit flies extended their lifespan [44]. This indicates the positive selection of this gene may be associated with the longer lifespan of great ape species compared with other primates [45] (Table S2).

**Figure 3:**
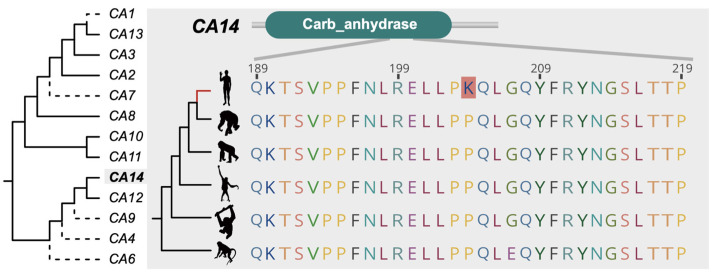
*CA14*, one of the copies from the carbonic anhydrase multigene family, is positively selected in the human lineage. Lys 204 was found to be under positive selection in *CA14* across all six primates. The gene phylogeny of the CA gene family in humans is shown on the left. The solid lines indicate genes present in all six primates and the dash lines indicate genes absent in some lineages. Animal illustrations are from phylopic.org, and are credited to NASA (*H. sapiens*, https://creativecommons.org/publicdomain/mark/1.0/, last accessed 23 July 2019), Gareth Monger (*P. abelii*, https://creativecommons.org/licenses/by/3.0/, last accessed 23 July 2019), Michael Keesey (*G. gorilla*, https://creativecommons.org/publicdomain/zero/1.0/, last accessed 23 July 2019), T. Michael Keesey and Tony Hisgett (*P. troglodytes*, https://creativecommons.org/licenses/by/3.0/, last accessed 23 July 2019).

We also found several PSGs that may contribute to great ape functional evolution. In humans, positive selection was detected in *CA14* at Lys 204 (Figure 3). Carbonic anhydrase (CA) is a large multigene family that contains 15 paralogs and is associated with reversible hydration of carbon dioxide in the primate. In this gene family, positive selection was only detected in one copy, *CA14*. CA14 catalyzes conversion between carbon dioxide and carbonic acid and bicarbonate ions in humans [46]. The *CA14* maintains a high expression level in the central nervous system in normal human adults [47] and *CA14* may also play an important role in modulating excitatory synaptic transmission in the brain [48], indicating that positive selection in *CA14* may contribute to the nervous system evolution in humans.

### 3.3. The Expression Pattern of Positively Selected Genes

**Figure 4:**
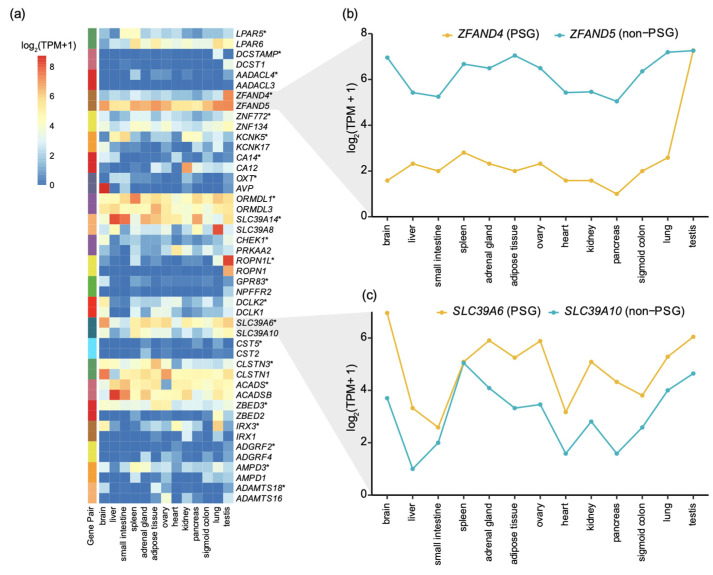
(**a**) Comparison of gene expression levels between PSGs and their closest paralogous non-PSGs. Twenty-three of thirty-nine PSGs in humans had the closest paralogous non-PSGs. The row annotation depicts the gene pair of PSG and its closest non-PSG. Asterisks (*) denote PSGs, and other genes are non-PSGs. Each PSG is next to its corresponding non-PSG. (**b**) Patterns of *ZFAND4* and *ZFAND5* expression in human tissues. (**c**) Patterns of *SLC39A6* and *SLC39A10* expression in human tissues.

We further investigated whether positive selection in humans affects the gene expression pattern in tissues by comparing PSGs in humans with their most similar paralogous copies in multigene families (Materials and Methods). Although the expression patterns between PSGs and their closest paralogous copies were different, we found that there are no uniform expression pattern shifts between these two copies, i.e., there is no clear correlation between the positive selection and tissue specificity or expression level (Figure 4 and Figure S3). For tissue specificity among the 23 gene pairs (PSG and its closest paralogous non-PSG), PSGs had higher tissue specificity than non-PSGs in 10 gene pairs (Figure S3a). For example, *ZFAND4* encodes zinc finger AN1-type-containing 4 and serves as a marker to predict metastasis and prognosis in oral squamous cell carcinoma [49], while *ZFAND4* was strictly expressed in the testis; its paralogous copy *ZFAND5* is a ubiquitously expressed gene and was highly expressed in other tissues such as the brain, lung, and testis (Figure 4b), indicating the genes not under positive selection harbor wide tissue and positive selection may eliminate the tissues ubiquity of *ZFAND4.* In the remaining 13 gene pairs, non-PSGs had higher tissue specificity than PSGs.

Among the expression levels of 23 gene pairs, the PSGs had higher mean expression levels across multiple tissues than non-PSGs in 11 gene pairs (Figure S3b). Taking the aforementioned *SLC39A6* as an example, *SLC39A6* (PSG) was expressed in all examined tissues (highest in brain), while its paralogous copy *SLC39A10* had a lower expression level across multiple tissues except in the spleen (Figure 4c). These results indicate that the association between selection and expression is divergent among different PSGs. Among the remaining 12 gene pairs, the non-PSGs had higher mean expression levels across multiple tissues than PSGs.

### 3.4. PSG and Disease Evolution

There was a hypothesis suggesting that PSGs tend to be associated with disease because the current environment of humans is substantially different from that of earlier hominins and thus previous adaptive mutations may become deleterious nowadays [50,51]. We tested this hypothesis using our newest PSGs and latest OMIM data [26]. In contrast to the results of the previous study [13] which found some support for the hypothesis, we found that there is no significant association between the human PSGs and human disease-associated genes (Table 6) and thus found no evidence to support that hypothesis.

## 4. Discussion

We detected 211 PSGs at all evolutionary branches in great apes. Compared with the previous study [13] using the same branch-site model [11], we identified 39 and 40 PSGs in humans and chimpanzees after filtering low-quality aligned sites and false positives caused by genetic drift, which ensure high confidence in PSGs. It should be noted that we could not identify all PSGs in all great ape evolution, especially those selected alleles that have not been fixed. Previous reports showed that the number of PSGs of chimpanzees is much greater than that of humans [13]. By contrast, we found that the great ape species experienced comparable numbers of positive selection. This could be caused by the update of annotation and assembly and our stricter filters for PSGs limiting the false positive PSGs and thus making the number of PSGs less than before, especially in chimpanzees whose genome then was poorly assembled.

Previous study showed that gene duplication results in relaxed selective constraint in one copy and is ultimately lost through pseudogenization in most cases [29]. However, we found that genes in multigene families, which experienced multiple rounds of gene duplication, can also be subject to positive selection, suggesting imbalanced positive selection resulted in the functional divergence of duplicates. We did not find a consistent expression pattern shift in PSGs and its closest paralogous copy (Figure 4 and Figure S3). We found positive selection led to a more non-uniform pattern across tissues. *SLC39A6* represents an interesting case given its high expression level in the human brain and its expression level is higher than its closest paralogous copy in multiple tissues. Previous studies showed that paralogs always show different expression profiles and are more tissue specific [52,53] and the evolutionary rate of a gene is negatively correlated with the expressional level [54,55]. This could be attributed to the fact that we only compared the expression level between PSG and non-PSG gene pairs instead of investigating the association between gene expression and evolutionary rate in all the genes [56], and thus this sampling led to different results. Interestingly, given that many PSGs are associated with functional and phenotypic changes in humans (e.g., the PSG *CA14* may be associated with metabolic evolution in humans), it would be fascinating to validate how these PSGs contribute to the phenotypic evolution in future studies.

We also found several genes are associated with aging. For example, *SOD1*, which plays important roles in senescence [41], experienced positive selection in the common ancestor of the great ape. Positive selection of this gene may be associated with the extended lifespan in great ape species compared with other primates (Table S2). Antagonistic pleiotropy (AP) hypothesis argues that the genes benefitting early life withstand more active positive selection [57], while these genes may impair late life and cause senescence. The pleiotropy role of this gene may be a case of AP hypothesis. Previous studies found that the AP hypothesis was supported by recently evolved enhancers in humans [58]. It will be interesting to test the AP hypothesis in coding regions based on population data in further studies. Unlike the previous study that found that PSGs in humans are enriched with human disease-associated genes [13], we found no evidence to support that conclusion in our analysis. This could be partly attributed to the update of genes in OMIM, which has grown from 847 in 2007 to 2301 nowadays, and the more stringent filters for PSGs we applied.

## 5. Conclusions

This study performed comparative evolutionary analysis of the latest assembled primate species to identify PSGs during great ape evolution. Among great ape species, the numbers of positively selected genes are comparable, even the differences in population size among these great apes should have resulted in differences in their ability to remove deleterious alleles and to adapt to changing environments. We also uncovered that more than half of multigene families exhibited signals of positive selection, suggesting that imbalanced positive selection resulted in the functional divergence of duplicates. We did not find a consistent shift in PSGs compared to their closest copies, implying that positive selection led to a more non-uniform pattern across tissues. PSGs in the human and Hominidae contribute to the neural transporter, antigens, and aging evolution. In addition, our results did not support the hypothesis that PSGs tend to be previously adaptive but deleterious nowadays.

## Figures and Tables

**Table 1:** Summary of assembly and annotation statistics for the primate genomes.

Species (Latin Name)	Genome Version	Genome Size (bp)	Gene Annotation Source	No. of Protein-Coding Gene
*Homo sapiens*	GRCh38.p13	3,272,089,205	NCBI *Homo sapiens* Updated Annotation Release 109.20210514	19,475
*Pan troglodytes*	Clint_PTRv2	3,024,031,013	NCBI *Pan troglodytes* Annotation Release 105	21,687
*Gorilla gorilla*	Kamilah_GGO_v0	3,044,872,214	NCBI *Gorilla gorilla* Annotation Release 102	20,784
*Pongo abelii*	Susie_PABv2	3,065,035,716	NCBI *Pongo abelii* Annotation Release 103	20,717
*Nomascus leucogenys*	Asia_NLE_v1	2,843,982,884	NCBI *Nomascus leucogenys* Annotation Release 103	20,656
*Macaca mulatta*	Mmul_10	2,971,331,530	NCBI *Macaca mulatta* Annotation Release 103	21,305

**Table 2:** Genetic positive selection of 14,397 one-to-one orthologs in great ape lineages.

Lineage	No. of PSG Candidates ^1^	No. of PSG Candidates without Potential Alignment Error	No. of PSG Candidates without Low Quality Aligned Columns	No. of PSGs without Relaxed Selection
*H. sapiens*	256	47	40	39
*P. troglodytes*	245	49	44	40
Hominini	65	11	11	11
*G. gorilla*	178	32	32	31
Homininae	80	40	39	39
*P. abelii*	181	38	35	34
Hominidae	55	22	20	20

^1^ *p*-value < 0.05 and with positively selected sites.

**Table 3:** Most of the PSGs were fixed in population.

Lineage	No. of PSGs	No. of Positively Selected Sites	No. of PSGs with Fixed Positively Selected Sites	No. of Fixed Positively Selected Sites
*H. sapiens*	39	111	31	91
*P. troglodytes*	40	133	40	129
Hominini	11	16	11	16
*G. gorilla*	31	76	31	75
Homininae	39	62	38	61
*P. abelii*	34	51	33	47
Hominidae	20	35	20	35

**Table 4:** Comparison of the PSGs identified by this study vs. Bakewell et al. [13].

Comparison		Human		Chimpanzee	
		This Study	Bakewell et al. [13]	This Study	Bakewell et al. [13]
	Shared	11		9	
Different Input	Different annotation versions	3	58	1	75
	Different ortholog assignment ^1^	-	16	-	22
	Problematic gene model in ortholog group ^2^	-	3	-	3
Different data processing method	No significance ^3^	-	25	-	96
	No significant sites ^4^	-	38	-	27
	positively selected sites with low confidence ^5^	-	3	-	1
	Newly detected PSGs in this study	25	-	30	-
Total		39	154	40	233

^1^ Six primate species, including two outgroup species in this study, versus three primate species, including one outgroup species in Bakewell’s study; ^2^ ortholog group contains genes with in-frame stop codons or whose CDS lengths are not a multiple of 3; ^3^ *p*-value < 0.05 by likelihood ratio test; ^4^ Bayes Empirical Bayes (BEB) posterior probability ≥ 0.95; ^5^ positively selected sites with alignment gaps or with alignment column score less than 1.

**Table 5:** Association of PSGs with multigene families.

Gene Type	No. of PSGs	No. of Non-PSGs	Proportion of PSGs	*p*-Value ^1^
* H. sapiens*				
No. of genes in multiple-gene family	23	10,793	0.002	0.992
No. of genes not in multiple-gene family	16	3565	0.004	
* P. troglodytes*				
No. of genes in multiple-gene family	33	10,783	0.003	0.186
No. of genes not in multiple-gene family	7	3574	0.002	
Hominini				
No. of genes in multiple-gene family	10	10,806	0.001	0.200
No. of genes not in multiple-gene family	1	3580	0.000	
* G. gorilla*				
No. of genes in multiple-gene family	25	10,791	0.002	0.317
No. of genes not in multiple-gene family	6	3575	0.002	
Homininae				
No. of genes in multiple-gene family	28	10,788	0.003	0.753
No. of genes not in multiple-gene family	11	3570	0.003	
* P. abeli*				
No. of genes in multiple-gene family	23	10,793	0.002	0.885
No. of genes not in multiple-gene family	11	3570	0.003	
Hominidae				
No. of genes in multiple-gene family	14	10,802	0.001	0.790
No. of genes not in multiple-gene family	6	3575	0.002	

^1^ Based on Fisher’s exact test.

**Table 6:** Association test of human PSGs with human disease.

Gene Type	No. of Disease Genes	No. of Non-Disease Genes	Proportion of Disease Genes	*p*-Value ^1^
PSGs	6	33	0.154	0.764
Non-PSGs	2690	11,668	0.187	

^1^ Based on Fisher’s exact test.

## Data Availability

The data described in this article are available in the article, its online Supplementary Materials, and public databases.

## Supplementary Materials

The following are available online at https://www.mdpi.com/article/10.3390/ani11113264/s1: Table S1: title: The PSGs across seven great ape lineages; Table S2 title: The comparison of the lifespan between great apes and other primates; Figure S1 title: An example of false positive selected genes caused by a poorly aligned region; Figure S2 title: Functional enrichment for PSGs in (a) the human and (b) the common ancestor of the great ape; Figure S3 title: Comparison of the expression pattern between the PSGs and non-PSGs.
