# Supplementary material for: Comparable Number of Genes Having Experienced Positive Selection among Great Ape Species
Source: Animals (Basel). 2021 Nov 15;11(11):3264. doi: 10.3390/ani11113264 (PMC8614513; doi:10.3390/ani11113264)
Supplement: Supplementary file 1 [file animals-11-03264-s001.zip › animals-1253659-supplementary.pdf]

Supplementary Material

for

Comparable number of genes having experienced positive selection among great ape species

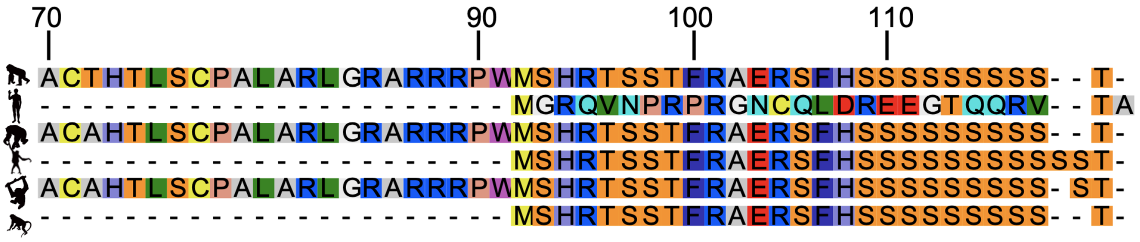

Figure S1. An example of false positive selected genes caused by a poorly aligned region. The 93th~110th amino acids in *HSPB7* was identified under positive selection in humans but alignments showed the neighbor regions of this site are poorly aligned, indicating this positive selected site may be false positive caused by the inconsistent isoform of different species. Thus, we removed this gene from PSG candidates.

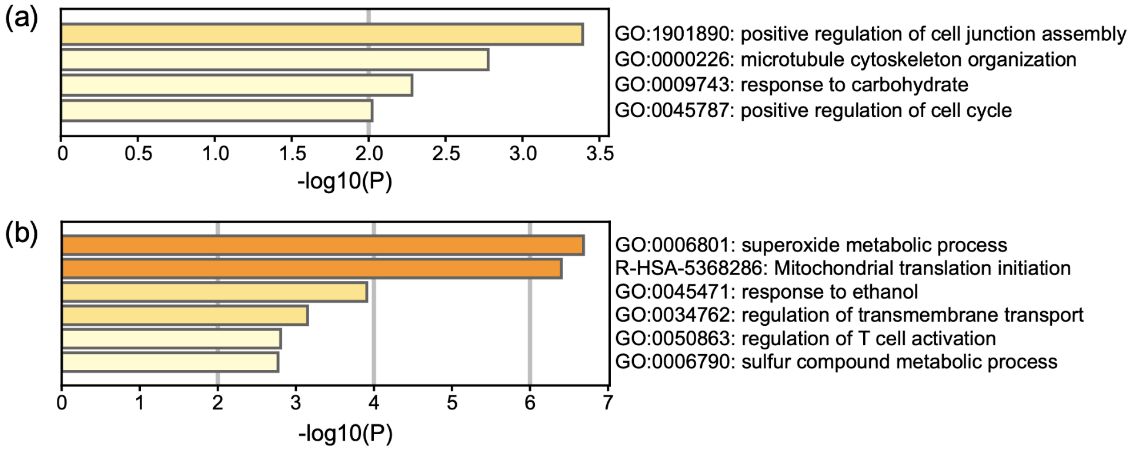

Figure S2. Functional enrichment for PSGs in (a) the human and (b) the common ancestor of the great ape.

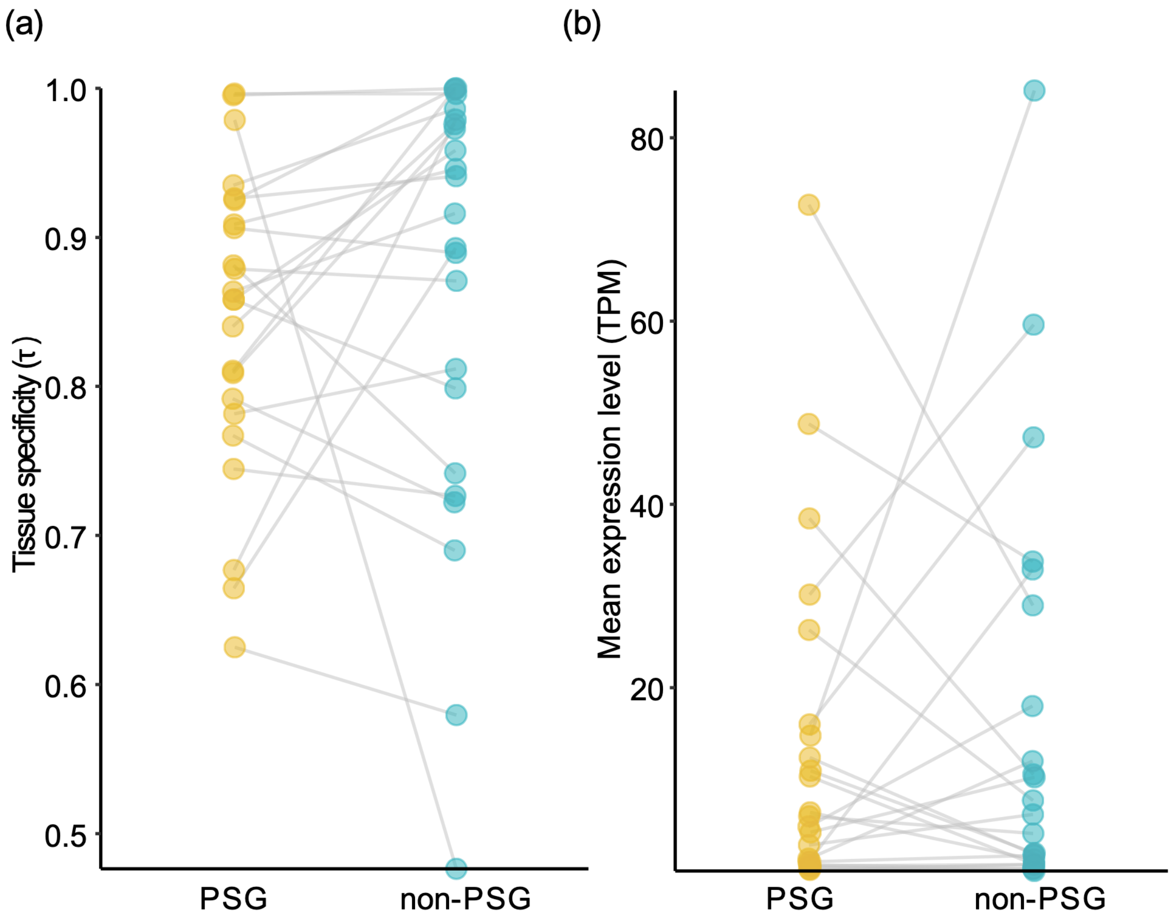

Figure S3. Comparison of the expression pattern between the PSGs and non-PSGs. (a) The comparison of tissue specificity between PSGs and non-PSGs. (b) The comparison of mean expression level across multiple tissues between PSGs and non-PSGs.

Table S1. The PSGs across seven great ape lineages.

Table S2. The comparison of the lifespan between great apes and other primates.

Species	Lifespan*	Source
Homo sapiens	122.5	The Anage database
Pan troglodytes	59.4	The Anage database
Gorilla gorilla	60.1	The Anage database
Pongo abelii	58	https://animaldiversity.org/accounts/Pongo_abelii/
Pongo pygmaeus	59	The Anage database

*The lifespan data of other 149 primates were downloaded from the AnAge database. To compare the lifespan between great ape and other primates, we performed bootstrap 1000 times and found the mean lifespan for great ape species was greater than other primates all the time.
